# Preparation of Human Kidney Progenitor Cultures and Their Differentiation into Podocytes

**DOI:** 10.21769/BioProtoc.4757

**Published:** 2023-08-20

**Authors:** Maria Elena Melica, Maria Lucia Angelotti, Giulia Antonelli, Anna J. Peired, Carolina Conte, Letizia De Chiara, Benedetta Mazzinghi, Elena Lazzeri, Laura Lasagni, Paola Romagnani

**Affiliations:** 1 Excellence Centre for Research, Transfer and High Education for the development of DE NOVO Therapies (DENOTHE), University of Florence, Florence, Italy; 2 Department of Experimental and Clinical Biomedical Sciences “Mario Serio”, University of Florence, Florence, Italy; 3 Nephrology and Dialysis Unit, Meyer Children’s Hospital IRCCS, Florence, Italy

**Keywords:** Kidney progenitor cells, Kidney, Podocytes, Tubular cells, Organoids, Chronic kidney disease

## Abstract

Kidney diseases are a global health concern. Modeling of kidney disease for translational research is often challenging because of species specificities or the postmitotic status of kidney epithelial cells that make primary cultures, for example podocytes. Here, we report a protocol for preparing primary cultures of podocytes based on the isolation and in vitro propagation of immature kidney progenitor cells subsequently differentiated into mature podocytes. This protocol can be useful for studying physiology and pathophysiology of human kidney progenitors and to obtain differentiated podocytes for modeling podocytopathies and other kidney disorders involving podocytes.

**Graphical overview**


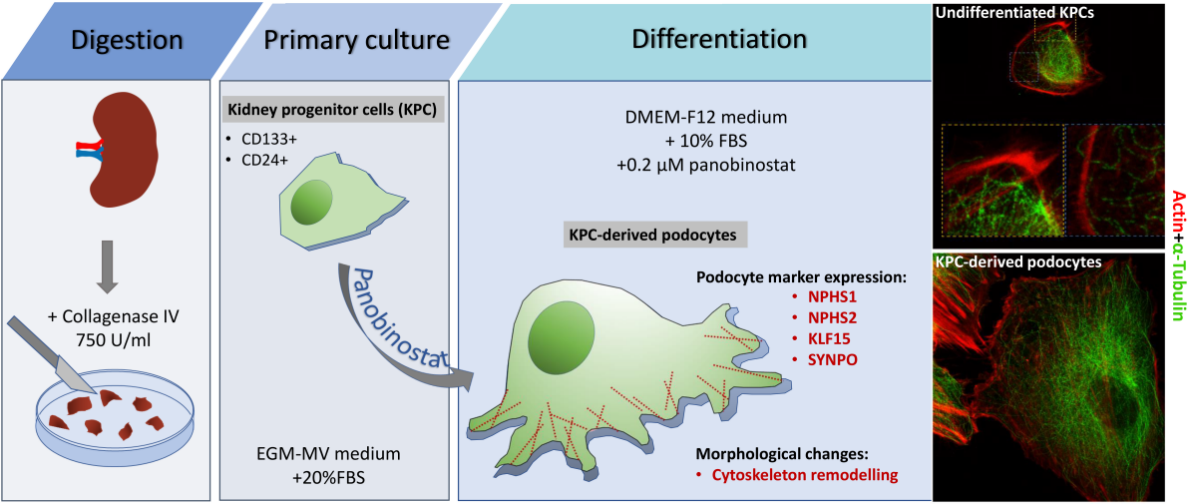


## Background

Kidney diseases, a global health issue, are the consequence of injury to the functional components of the kidney, the nephrons (Romagnani et al., 2017). Nephrons are constituted by a blood filtering unit, the glomerulus, and the respective tubule where the filtrate is modified by solute reabsorption and metabolite secretion up to when the final urine is excreted via the urinary tract (Romagnani et al., 2017). The nephrons respond to injury in two ways: a) differentiated epithelial cells undergo polyploidization and hypertrophy to rapidly support residual kidney function and b) immature epithelial cells, referred to as kidney progenitors (Lazzeri et al., 2019), proliferate to recover at least a part of the lost cells, i.e., kidney regeneration (Lazzeri et al., 2019). Kidney progenitors are localized along the inside of the Bowman capsule of the glomerulus and are scattered among tubular epithelial cells along the tubule, being identified by the expression of the surface markers CD133 and CD24, in humans (Sagrinati et al., 2006; Lazzeri et al., 2019). Kidney progenitors can be obtained from kidney tissue or urine and cultured long term (Angelotti et al., 2012) because they retain the capacity for self-renewal. Kidney progenitors have the capacity to differentiate into multiple types of kidney epithelial cells in vitro and in vivo (Sagrinati et al., 2006; Lazzeri et al., 2019). Hence, kidney progenitors can be expanded and differentiated into different types of tubular epithelial cells (Angelotti et al., 2012) and even cultured in 3D, generating tubuloids, a selective property among other kidney tubular cells (Xu et al., 2022). This property makes them ideal for modeling of genetic tubular disorders, e.g., upon isolation from the urine of patients with genetic tubular disorders or upon introduction of pathogenic genetic variants (e.g., using CRISPR-Cas system) (Xu et al., 2022). In addition, kidney progenitors can be differentiated in culture into podocytes, the main constituent of the glomerular filtration barrier. Podocytes are highly differentiated postmitotic cells unable to proliferate (Kopp et al., 2020). For this reason, they are impossible to expand in primary cultures, unless using artificial systems of immortalization (Shankland et al., 2007).

Here, we report detailed protocols on how to prepare human kidney progenitor cultures from human kidney tissue, maintain them, and differentiate them into podocytes. Differentiation of kidney progenitors using specific factors and compounds (Lasagni et al., 2015), as recently reported for the histone deacetylase inhibitor panobinostat, induces a change in their phenotype, promoting transcription of podocyte genes such as nephrin, podocin, and synaptopodin (Melica et al., 2022). We also report methods to assess their phenotype by qRT-PCR, FACS, and confocal and stimulated emission depletion (STED) microscopy. Applying the same culturing method described here to the isolation procedure reported by Lazzeri et al. (2015) permits the preparation of kidney progenitor cultures also from the urine of patients with kidney disorders, making them particularly suitable for studying genetic podocytopathies for diagnostic purposes. Given the importance of kidney progenitors and podocytes in the pathogenesis of chronic kidney disease, the possibility to prepare and maintain these cultures has wide implications and possible uses.

## Materials and reagents

**Biological materials**

Normal-appearing kidney fragments are obtained from the pole opposite to the tumor from patients that underwent nephrectomy for localized renal tumors.

**CAUTION:** All the experiments involving human specimens should be performed in accordance with the recommendations of the Institutional Ethical Committee for human experimentation. All procedures in this protocol were conducted under protocols approved by the Ethical Committee on human experimentation of the Careggi University Hospital.

**Reagents**

1. Physiological saline solution, NaCl 0.9% (B. Braun Melsungen AG, A.I.C. n. 030902391)
2. HyClone defined fetal bovine serum (FBS), US origin (Cytiva, catalog number: SH30070.03)
3. Endothelial cell growth basal medium (EBM), 500 mL (LONZA, catalog number: CC-3121)
4. Microvascular endothelial growth medium (EGM-MV) SingleQuots kit (LONZA, catalog number: CC-4123) [contains growth factors, cytokines, and supplements: bovine brain extract w/o heparin (BBE); hydrocortisone (hEGF); gentamicin, amphotericin B (GA-1000); and FBS]
5. DMSO (Merck KGaA, catalog number: D8418)
6. Dulbecco’s modified Eagle’s medium/Ham’s nutrient mixture F-12 (DMEM-F12-1L) (Merck KGaA, catalog number: D2906)
7. Collagenase type IV (Sigma, catalog number: C-5138)
8. Panobinostat, LBH589 (MedChem Express, catalog number: HY-10224)
9. Trypsin/EDTA solution 0.025% (LONZA, catalog number: CC-5012)
10. Sodium hydrogen carbonate (NaHCO_3_) (Merck KGaA, catalog number: S5761)
11. Bi-distilled water (ddH_2_O)
12. D-PBS, no calcium, no magnesium (Gibco, catalog number: 14190-094)
13. Ammonium chloride (NH_4_Cl) (Carlo Erba, catalog number: 419416, CAS number: 12125-02-9)
14. FcR blocking reagent human (Miltenyi Biotec, catalog number: 130-059-901)
15. Bovine serum albumin (BSA) (Merck KGaA, catalog number: A9747)
16. Sodium azide (NaN_3_) (Merck KGaA, catalog number: S2002, CAS number: 26628-22-8)
17. RNeasy Micro kit (Qiagen, catalog number: 74004)
18. TaqMan reverse transcription reagents (Invitrogen, catalog number: N8080234)
19. TaqMan Fast Universal PCR Master Mix (2×), no AmpErase^TM^ UNG (Applied Biosystems, catalog number: 4352042)
20. Human GAPD (GAPDH) endogenous control (VIC^TM^/TAMRA^TM^ probe, primer limited) (Applied Biosystems, catalog number: 4310884E)
21. TaqMan Gene Expression Assay Mix (20×) (Table 1)
  **Table 1.** TaqMan assay list

Gene symbol	Gene name	TaqMan assay ID
NPHS1	NPHS1, nephrin	Hs00190446_m1
NPHS2	NPHS2, podocin	Hs00387817_m1
SYNPO	Synaptopodin	Hs00200768_m1
KLF15	Kruppel like factor 15	Hs00362736_m1
22. Paraformaldehyde solution 4% in PBS (PFA) (Santa Cruz, catalog number: sc-281692)
23. Triton X-100 (Merck KGaA, catalog number: X100RS-SG)
24. 4’,6-Diamidino-2-phenylindole (DAPI) (Merck KGaA, catalog number: D9542)
25. Goat serum (Vector Laboratories, catalog number: S-1000)
26. Donkey serum (Merck KGaA, catalog number: D9663)
27. Antibodies used for FACS assay and immunofluorescence (Table 2)
  **Table 2.** Antibodies list

Antibody	Vendor	Catalog number	Final concentration
**Primary antibody**			
CD133/2	Miltenyi Biotec	130-090-851	10 μL/test
CD24 (clone SN3)	Santa Cruz	SC-19585	10 μg/mL
mouse IgG1	Miltenyi Biotec	130-106-545	10 μg/mL
mouse IgG2b	Miltenyi Biotec	130-106-547	10 μg/mL
α-Tubulin	Merck KGaA	T6074	2 μg/mL
Sir-Actin	Spirochrome	SC001	1 μM
Nephrin (NPHS1)	R&D system	AF4269	4 μg/mL
Podocin (NPHS2)	Abcam	ab50339	30 μg/mL
**Secondary antibody**			
Goat anti-mouse IgG1-Alexa Fluor 488	Molecular Probes	A-21121	6 μg/mL
Goat anti-mouse IgG2b-Alexa Fluor 647	Molecular Probes	A-21242	6 μg/mL
Goat anti-mouse IgG1-Alexa Fluor 594	Molecular Probes	A-21125	20 μg/mL
Donkey anti-sheep IgG(H+L)-Alexa Fluor 488	Molecular Probes	A-11015	2 μg/mL
Goat anti-rabbit IgG(H+L)-Alexa Fluor 546	Molecular Probes	A-11035	2 μg/mL
28. Kidney progenitor cell growth medium (see Recipes)
29. Kidney progenitor cell washing medium (see Recipes)
30. Red blood cell lysis buffer (NH_4_Cl 0.08%) (see Recipes)
31. Freezing medium (see Recipes)
32. DMEM-F12 + 10% HyClone defined FBS (see Recipes)
33. FACS buffer (PBS 1× 0.5% BSA - 0.02% NaN3 buffer) (see Recipes)

## Equipment

1. Pipettes
2. Vacuum filtration system with 0.22 μm cellulose acetate (CA) membrane, 500 mL filters (Corning, catalog number: 430769).
3. Sharp forceps, straight (2-biol, catalog number: 91156-11)
4. Sterile plates 100 mm × 20 mm (Corning, catalog number: 430167)
5. Cell dissociation sieves (Merck KGaA, catalog number: S1145)
6. Screen for cell dissociation, size 80 mesh screens (Merck KGaA, catalog number: S3770)
7. Screen for cell dissociation, size 60 mesh screens (Merck KGaA, catalog number: S1020)
8. Glass pestle
9. Ice bucket
10. 6-well clear TC-treated multiple well plates (Corning, catalog number: 3516)
11. Cryogenic vial (Corning, catalog number: CC430659)
12. Freezing container (Thermo Scientific, catalog number: 5100-0001)
13. 75 cm^2^ flask (Corning, catalog number: CC430641)
14. Polypropylene urine container, 120 mL (Biosigma, catalog number: BSC258)
15. Serological pipettes 2 mL (Corning, catalog number: CLS4486)
16. Serological pipettes 5 mL (Corning, catalog number: CLS4487)
17. Serological pipettes 10 mL (Corning, catalog number: CLS4488)
18. 15 mL tube (Corning, catalog number: 430791)
19. 50 mL tube (Corning, catalog number: 430829)
20. 1.5 mL microcentrifuge tubes (Axygen, catalog number: MCT-150-C-S)
21. 0.2 mL RNase-free PCR tubes (Invitrogen, catalog number: AM12225)
22. MicroAmp fast optical 96-well reaction plate with barcode (Applied Biosystems, catalog number: 4346906)
23. MicroAmp optical adhesive film (Applied Biosystems, catalog number: 4360954)
24. GeneExplorer thermal cycler 96 × 0.2 mL (Bioer, catalog number: GE-96G)
25. 7900HT Fast Real-Time PCR system (Applied Biosystems, catalog number: 4351405)
26. Centrifuge with plate holders
27. Inverted phase contrast microscope (Zeiss, Z-AXIO40C)
28. Heracell 150i CO_2_ incubator
29. Bürker counting chamber (Merck KGaA, catalog number: BR719505-1EA)
30. Flow cytometer (Miltenyi Biotec, MacsQuant Analyzer)
31. 2-well chamber slide coverslip (Nunc Lab-Tek II, catalog number: 155379PK)
32. Confocal microscope (Leica Microsystems, LEICA SP8 STED 3X confocal microscope)
33. Biological safety cabinet (Angelantoni Life Science Srl, catalog number: CTH48C2)
34. 4 °C fridge, -20 °C freezer, and -80 °C freezer

## Software

1. Flow Cytometry Analysis software (Inivai, Flowlogic software)
2. Confocal microscope acquisition software Las X (Leica Microsystems)
3. Huygens Professional software version 18.04 (Scientific Volume Imaging B.V.)

## Procedure

- **Human kidney progenitor cells (KPC): isolation, maintenance, and cryopreservation**
  In this section, we describe how to isolate primary kidney progenitor cells from human tissue. The method we describe exploits the ~50-fold higher proliferative capacity of KPC cells in comparison to other renal cell types in a specific growth medium (Peired et al., 2020). Based on our experience, this method allows to obtain a pure population of viable kidney progenitors more easily than the multi-step process based on separation using magnetic beads.
  1. Isolation of KPC from human kidney tissue
    - Collect a fragment of kidney cortex (from 1 to 3 cm^3^) from the pole opposite to the tumor. Store the tissue in sterile physiological saline solution during transport to the laboratory. We recommend performing the kidney cell isolation within 1 h after surgical tissue collection.
    - Remove the kidney capsule and transfer the sample to a 100 mm sterile dish. Mince the cortex in pieces as small as possible using a scalpel. Add to the dish 5 mL of 750 U/mL collagenase type IV prepared in EBM medium. Incubate for 45 min at 37 °C in the incubator. Neutralize the enzymatic reaction by adding 10 mL of EBM containing 10 % FBS. **CAUTION:** During mincing, maintain the tissue fragments humidified by adding a drop of sterile physiological saline solution.
    - Transfer the suspension to graded mesh screens (60 and 80 mesh). Mechanically break down the tissue suspension using a glass pestle and pass it through the 60 and 80 mesh screens. Wash thoroughly with 20 mL of kidney progenitor cell washing medium and recover the flowthrough in a polypropylene urine container. Transfer this suspension to a 50 mL polypropylene tube and centrifuge at 400× *g* for 5 min at 4 °C.
    - Aspirate the supernatant and wash the pellet with 5 mL of PBS. Centrifuge at 400× *g* for 5 min at 4 °C. Discard the supernatant and resuspend the pellet in 5 mL of red blood cell lysis buffer.
    - Incubate for 4 min at 37 °C and then stop the reaction by adding 10 mL of kidney progenitor cell washing medium.
    - Centrifuge at 400× *g* for 5 min at 4 °C.
    - Remove supernatant and resuspend the pellet in 10 mL of kidney progenitor cell growth medium.
    - Count cell suspension using a Bürker counting chamber.
    - Transfer the cells in 75 cm^2^ flask (500,000 cells/flask) in 8 mL/flask kidney progenitor cell growth medium. Label as passage 0.
    - Place the cells in a 5% CO_2_, 37 °C incubator.
    - After three days, replace the medium with fresh kidney progenitor cell growth medium to remove unattached cells and continue with twice-a-week changes until cells reach 80% confluency. It usually takes 7–10 days.
  2. Expansion and sub-culturing of KPC
    - Sub-culture when the cells are approximately 80% confluent.
    - Aspirate the medium and wash with 6 mL of PBS.
    - Aspirate PBS and add 2 mL of a 0.25 mg/mL trypsin/EDTA solution. Incubate in a 37 °C incubator for approximately 5 min. Check under the microscope if cells are detached.
    - Neutralize the enzymatic reaction by adding 4 mL of kidney progenitor cell washing medium and collect the cells in a 15 mL polystyrene tube. Centrifuge the cells at 400× *g* for 5 min at 4 °C.
    - Aspire supernatant without disturbing the cell pellet and resuspend the cells in 5 mL of kidney progenitor cell growth medium.
    - Count the number of cells using a Bürker counting chamber.
    - Replate the cells in 75 cm^2^ flasks with a ratio of 1:3 in kidney progenitor cell growth medium.
    - Change medium twice a week during maintenance of cultures in a 5% CO_2_, 37 °C incubator.
  3. Cryopreservation of KPC
    - Kidney progenitors are cryo-stored in 1 mL of freezing medium at a density from 5 × 10^5^ up to 1 × 10^6^ cells/cryogenic vial.
    - Aspirate the medium from the 75 cm^2^ flask and wash with 6 mL of PBS.
    - Aspirate the PBS and add 2 mL of a 0.25 mg/mL trypsin/EDTA solution. Incubate in a 37 °C incubator for approximately 5 min. Check under the microscope if cells are detached.
    - Neutralize the enzymatic reaction by adding 4 mL of kidney progenitor cell washing medium and collect the cells in a 15 mL polystyrene tube. Centrifuge the cells at 400× *g* for 5 min at 4 °C.
    - Aspire supernatant without disturbing the cell pellet and resuspend the cells in 5 mL of kidney progenitor cell growth medium.
    - Count the number of cells using a Bürker counting chamber.
    - Centrifuge the cells at 400× *g* for 5 min at 4 °C.
    - Resuspend the cell pellet in freezing medium at a density of 1 × 10^6^ cells/mL. Mix well.
    - Aliquot in 1 mL per cryovial.
    - Transfer the cryovials to a freezing container and put the freezing container into a -80 °C freezer.
    - The next day, transfer the cryovials to a liquid nitrogen tank for long-time storage. **CAUTION:** Minimize as much as possible the time cells remain in freezing medium at room temperature. Transfer immediately in the freezing container to -80 °C.
  4. Thawing of kidney progenitor cells
    - Thaw a vial of cells. To achieve rapid warming, place the frozen vial into a 37 °C water bath.
    - Transfer immediately the content of each vial to a 15 mL tube containing 4 mL of kidney progenitor cell washing medium.
    - Centrifuge at 400× *g* for 5 min at 4 °C.
    - Remove the supernatant and resuspend the cells into 2 mL of kidney progenitor cell growth medium.
    - Transfer the cell suspension to 75 cm^2^ flasks at a density of approximately 500,000 cells/flask in 8 mL/flask of kidney progenitor cell growth medium. Place the cells in a 5% CO_2_, 37 °C incubator.
- **Differentiation of KPC into podocytes**
  1. Detach the cells at 60%–80% confluency with trypsin as described above.
  2. Count cells and plate in a 6-well plate at a density of 80,000 cell/well in 1.5 mL/well of kidney progenitor cell growth medium.
  3. Place the cells in a 5% CO_2_, 37 °C incubator.
  4. After 5–6 h (or when cells are attached to the plate), gently remove the medium and replace with 1.5 mL/well of EBM without any supplement and without serum. Place the cells in a 5% CO_2_, 37 °C incubator.
  5. After 16 h, remove the EBM medium and stimulate the cells for 48 h with 1.5 mL/well of differentiation medium containing 0.2 μM panobinostat in DMEM-F12 + 10% HyClone Defined FBS.
  6. At the end of differentiation, characterize the cells using qRT-PCR and immunofluorescence. **CAUTION:** To obtain better differentiation results, use cells at early passages (P1–P2).

## Data analysis

- **Flow cytometry analysis for purity check**
  It is important to characterize each primary kidney progenitor cell culture, evaluating in the various passages (from passage P0 to at least passage P3) the expression of surface markers CD133 and CD24. To evaluate CD133 and CD24 expression, perform flow cytometry analysis as reported:
  1. Detach the cells with trypsin as described above.
  2. Count cells.
  3. Prepare two 1.5 mL tubes: label one *Isotype control* and the other *Antibody* (CD133 and CD24).
  4. Transfer 100,000 cells in each tube.
  5. Centrifuge at 400× *g* for 5 min at 4 °C.
  6. Aspirate supernatant without disturbing the cell pellet. Add 5 μL of FcR blocking reagent human on cell pellet.
  7. Prepare the staining solutions as follows:
    - Isotype control mix I: Add 1 μL of mouse IgG2b (to obtain a final concentration of 10 μg/mL) and 1 μL of mouse IgG1 (to obtain a final concentration of 10 μg/mL) in 98 μL of FACS buffer.
    - Antibody mix I: Add 10 μL of CD133/2 (10 μL/test) antibody and 2 μL of CD24 (to obtain a final concentration of 10 μg/mL) antibody in 28 μL of FACS buffer.
  8. Add 30 μL of the staining solutions to the corresponding tube containing cells and FcR blocking reagent human. Resuspend the pellet.
  9. Incubate on ice for 15–30 min covered with a tin foil.
  10. Add 500 μL of FACS buffer.
  11. Centrifuge at 400× *g* for 5 min at 4 °C and aspirate the supernatant without disturbing the cell pellet.
  12. Resuspend each pellet in 30 μL of the staining solution II, prepared as follows:
    Staining Solution II: add 1 μL of goat anti-mouse IgG2b-647 (to obtain a final concentration of 5 μg/mL) and 1 μL of goat anti-mouse IgG1-488 (to obtain a final concentration of 5 μg/mL) to 300 μL of FACS buffer.
  13. Incubate on ice for 15–30 min covered with a tin foil.
  14. Add 500 μL of FACS buffer.
  15. Run FACS assay by using MacsQuant Analyzer.
  16. Analyze the FACS data using Flowlogic software. A representative FACS assay for kidney progenitor cells is shown in Figure 1. **CAUTION:** Use primary cultures consisting of at least 95% of CD133 and CD24 double-positive cells. The percentage of double-positive cells tend to increase during the first two passages because the kidney progenitor cell growth medium allows the selective growth of undifferentiated kidney progenitors.
    **Figure 1.**
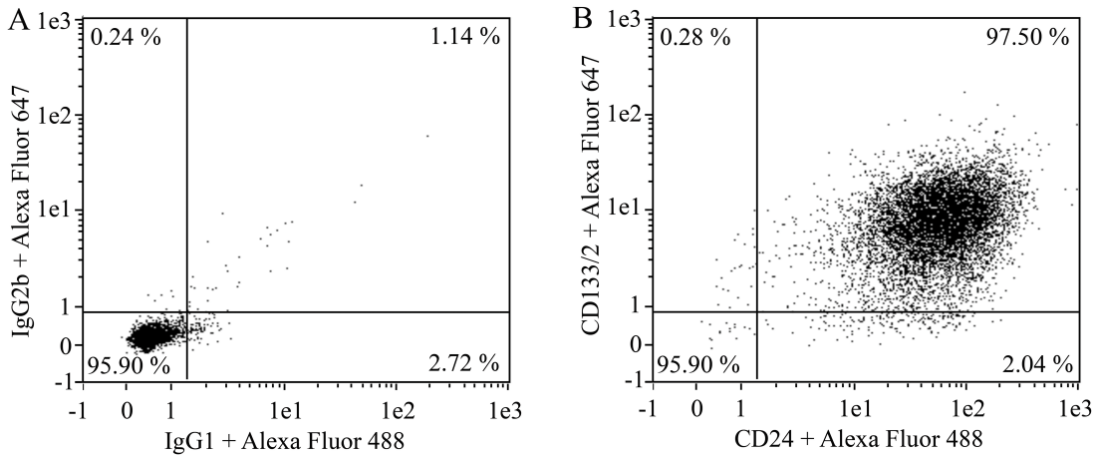
Evaluation of CD133/2 and CD24 expression in kidney progenitor cells by flow cytometry. Representative flow cytometry dot plot graphs showing the percentage of CD133 and CD24 positive cells in primary kidney progenitor cells at passage P1 (B). Staining of the same cells with isotype control antibodies is shown in (A).
- **Evaluation of differentiation**
  After 48 h of stimuli with differentiation medium, it is possible to evaluate differentiation status of the cells by using qRT-PCR and immunofluorescence (Figure 2).
  **Figure 2.**
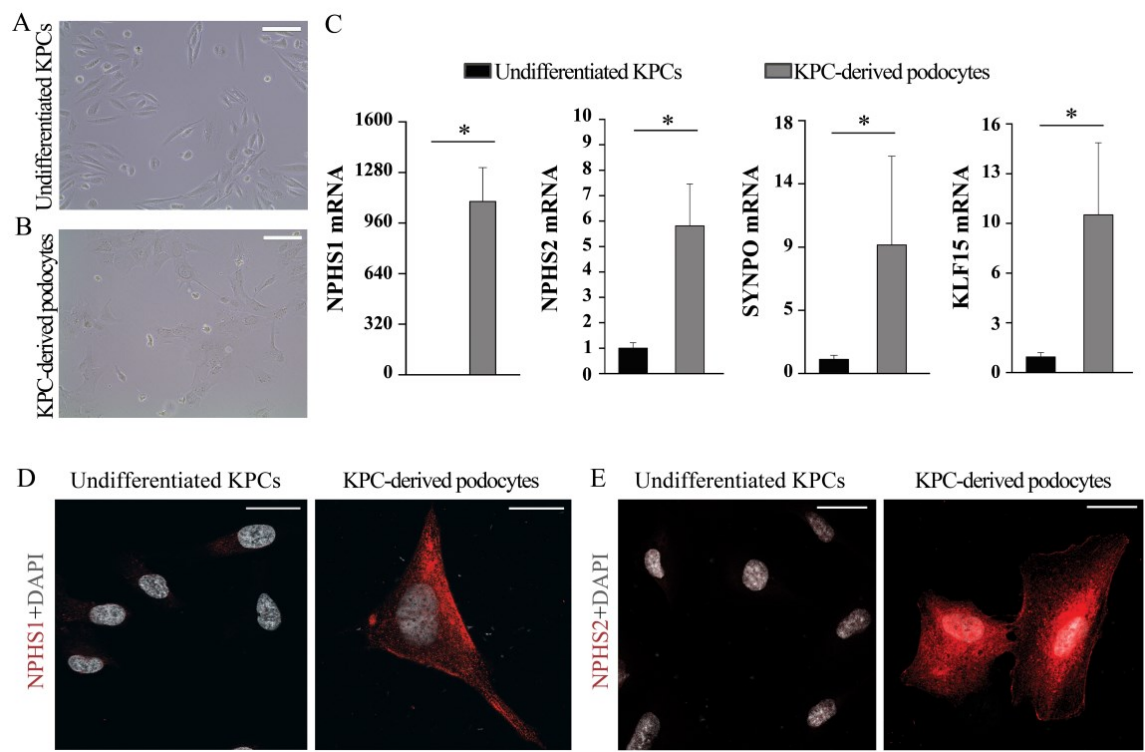
Evaluation of podocytes derived from kidney progenitor cells. Representative phase contrast images of (A) undifferentiated kidney progenitor cells and (B) podocytes derived from kidney progenitor cells after 48 h of differentiation. Scale bars, 100 μm. (C) qRT-PCR assay of the podocyte markers NPHS1, NPHS2, KLF15, and SYNPO in undifferentiated kidney progenitor cells and in podocytes derived from kidney progenitor cells. mRNA expression of the podocytes markers was determined by qRT-PCR and reported as mean ± SEM of fold increase over undifferentiated cells. (D, E) Representative confocal microscopy images showing expression of the podocyte markers NPHS1 and NPHS2 (red) in undifferentiated kidney progenitor cells and in podocytes derived from kidney progenitor cells. DAPI (white) was used to counterstain nuclei. Scale bars, 25 μm. KPC, kidney progenitor cell.
- **qRT-PCR assay**
  One of the methods to check the differentiation status of the cells is to use real-time PCR assay to evaluate mRNA expression level of podocyte markers, such as NPHS1, NPHS2, KLF15, and SYNPO. We perform qRT-PCR for each marker on the RNA extracted from an equal number of undifferentiated and differentiated cells independently from the RNA concentration obtained. The qRT-PCR protocol is detailed below:
  1. Collect pellets from 100,000 undifferentiated and differentiated cells and extract RNA using the RNeasy Micro kit, followed by the DNase digestion protocol in a final volume of 15 μL per sample.
  2. Proceed to the synthesis of the cDNA using the TaqMan reverse transcription reagents:
    - Prepare the following cDNA synthesis mix on ice in a 0.2 mL PCR tube. Mix thoroughly and centrifuge briefly.
      **Table:** 

Component	Volume for reaction
10× buffer	5 μL
25 mM MgCl_2_	3.5 μL
dNTPs	10 μL
RNase inhibitor	2.5 μL
Random hexamers	2.5 μL
Multiscribe (MULV)	2.5 μL
RNA	15 μL
RNase-free water	To 50 μL
  3. Proceed with the following incubation protocol in a thermal cycler.
    **Table:** 

Step	Temperature	Run time
1	25 °C	10 min
2	48 °C	30 min
3	95 °C	5 min
4	4 °C	Hold
  4. Collect cDNA synthesis product for qPCR or store in a -20 °C freezer.
  5. Prepare the qPCR mix for each podocyte gene (NPHS1, NPHS2, KLF15, or SYNPO) on ice by adding the components below. Mix extra 10% for more reactions. Mix thoroughly and centrifuge briefly.
    **Table:** 

Component	Volume for one reaction
TaqMan Fast Universal PCR Master Mix (2×)	10 μL
TaqMan Assay (NPHS1, NPHS2, KLF15, or SYNPO)	1 μL
ddH_2_O	4 μL
Final volume	15 μL
  6. Prepare the qPCR mix for GAPDH housekeeping gene on ice by adding the components below. Mix extra 10% for more reactions. Mix thoroughly and centrifuge briefly.
    **Table:** 

Component	Volume for one reaction
TaqMan Fast Universal PCR Master Mix (2×)	10 μL
GAPDH endogenous control	1 μL
ddH_2_O	8 μL
Final volume	19 μL
  7. Dispense 15 μL of the mix for NPHS1, NPHS2, KLF15, or SYNPO into each well of a 96-well PCR plate. For GAPDH, dispense 19 μL of the mix.
  8. Add 5 μL of cDNA samples to the well containing the mix for NPHS1, NPHS2, KLF15, or SYNPO and add 1 μL of cDNA samples to the well containing the mix for GAPDH.
  9. Seal the plate and centrifuge briefly. Proceed to the incubation below by selecting fast mode:
    **Table:** 

Step	Temperature	Run time
1	95 °C	20 s
2	95 °C	1 s
3	60 °C	20 s + data collection
4	Repeat steps C2–C3 40 times
  10. Perform data analysis: gene expression of each marker is normalized to that of the GAPDH. For each marker, results are reported as fold change of expression of the differentiated cells over undifferentiated cells (Figure 2).
- **Immunofluorescence**
  A differentiative program induces morphology changes and leads to cell-specific protein expression that can be evaluated by immunofluorescence assay. The differentiation of kidney progenitor cells into podocytes is confirmed on the basis of the NPHS1 and NPHS2 podocyte marker expression (Figure 2), while the tubulin and actin expression assessed by super-resolution microscopy shows the drastic cytoskeleton changes associated with differentiation (Figure 3). The immunofluorescence procedure is detailed below:
  1. Detach the cells with trypsin as described above.
  2. Count cells.
  3. Plate cells onto a 2-well chamber slide at a density of 20,000 cell/well in 1 mL/well of kidney progenitor cell growth medium.
  4. Place the cells in a 5% CO_2_, 37 °C incubator.
  5. After 5–6 h (or when cells are attached to the plate), gently remove the medium and replace with 1.5 mL/well of EBM without any supplement and without serum. Place the cells in the incubator with 5% CO_2_ and 37 °C.
  6. After 16 h, remove the EBM medium and stimulate cells for 48 h with 1 mL/well of 0.2 μM panobinostat in DMEM-F12 + 10% HyClone defined FBS.
  7. At the end of the differentiation, remove chamber slides from the incubator.
  8. Aspirate the medium and wash the cells with 500 μL/chamber of PBS.
  9. Add 1 mL/chamber of 4% PFA and incubate for 20 min at room temperature.
  10. Gently wash the slides three times with 1 mL of PBS.
  11. Add permeabilizing solution (composed of 0.5% Triton X-100 in PBS) if required from the antibody user manual (antibody information is reported in Table 2 and Table 3) for 5 min at room temperature.
    **Table 3.** Immunofluorescence details

Primary antibody		Secondary antibody			
**Antibody**	**Final concentration**	**Antibody**	**Final concentration**	**Permeabilization**	**Blocking serum required**
α-Tubulin	2 μg/mL	Goat anti-mouse IgG1-Alexa Fluor 594	20 μg/mL	Required	Goat
Sir-Actin	1 μM	/	/	Required	/
Nephrin (NPHS1)	4 μg/mL	Donkey anti-sheep IgG(H+L)-Alexa Fluor 488	2 μg/mL	Not required	Donkey
Podocin (NPHS2)	30 μg/mL	Goat anti-rabbit IgG(H+L)-Alexa Fluor 546	2 μg/mL	Required	Goat
  12. Wash for 5 min with PBS.
  13. Incubate with blocking solution containing 3% BSA and 0.3% serum (goat or donkey, as reported in Table 3) in PBS.
  14. Remove blocking solution without washing.
  15. Incubate with primary antibody (Table 3) for 15 min at 37 °C and subsequently for 1 h at 4 °C covered with a tin foil.
  16. Wash for 5 min with PBS.
  17. Incubate with the secondary antibodies listed in Table 3 and with 1 μg/mL DAPI in PBS 1× for 30 min at room temperature covered with a tin foil to block the light.
  18. Acquire images using a LEICA SP8 STED 3X confocal microscope.
  19. For STED analysis, frame sequential acquisition can be applied to avoid fluorescence overlap. A 775 nm pulsed-depletion laser was used and a gating between 0.3 and 6 ns was applied to avoid collection of reflection and autofluorescence. Images were acquired with Leica HC PL APO CS2 100×/1.40 oil STED white objective. De-convolve with Huygens Professional software (Romoli et al., 2018).
  **Figure 3.**
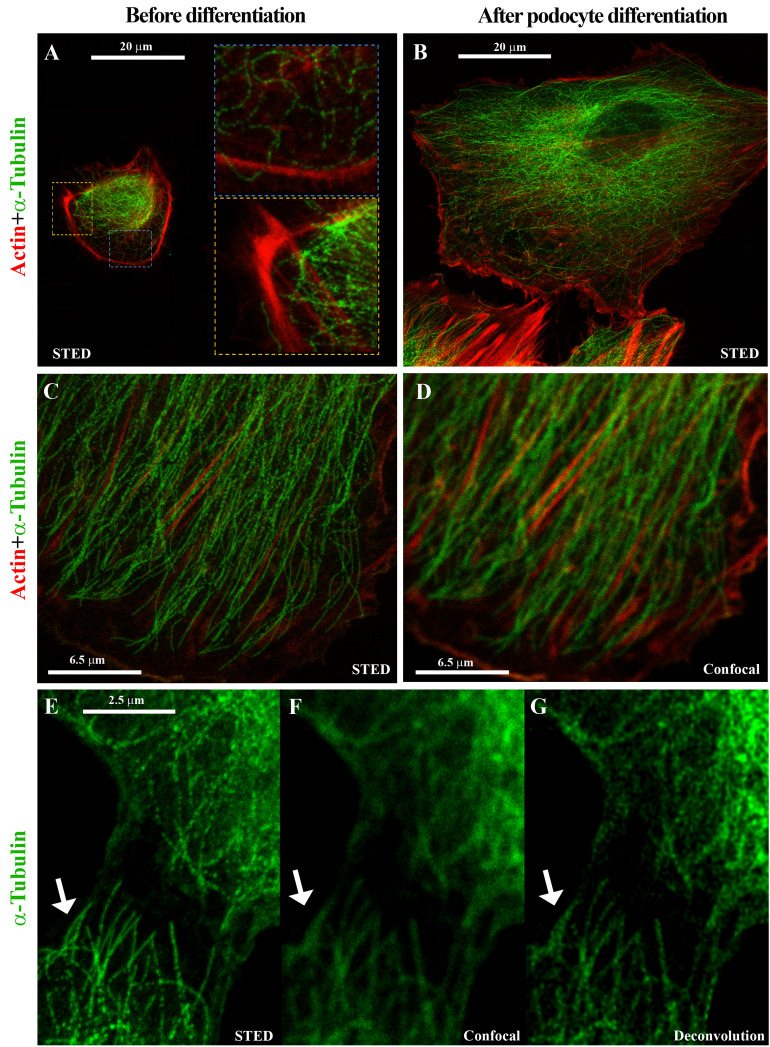
Stimulated emission depletion (STED) super-resolution microscopy shows cytoskeleton changes associated with differentiation. Representative STED images showing cytoskeleton changes associated with differentiation into podocytes based on α-Tubulin (green) and Actin (red) expression in primary human kidney progenitor cells before (A) and after 48 h differentiation (B–G). Compared to confocal microscopy (D, F), the use of STED microscopy and deconvolution software allows to identify the cytoskeleton organization with nanoscopic spatial resolution (C, E, and G).

## Recipes

1. **Kidney progenitor cell growth medium (EGM-MV medium + 20% HyClone defined FBS)**
  The renal progenitor cells are grown in EGM-MV medium supplemented with 20% HyClone defined FBS, prepared as follows:
  Supplement 400 mL of EBM medium with BBE and hEGF provided in the microvascular endothelial growth medium (EGM-MV) SingleQuots kit and with 100 mL of HyClone defined FBS. Filter using a vacuum filtration system with 0.22 μm CA membrane and store at 4 °C.
2. **Kidney progenitor cell washing medium (EBM medium + 10% FBS)**
  Supplement EBM medium with 10% FBS by using the serum provided with the SingleQuots kit (and not used for EGM-MV + 20% HyClone formulation). Other commercial FBS can be used. Filter the solution using a vacuum filtration system with 0.22 μm CA membrane.
3. **Red blood cell lysis buffer (NH_4_Cl 0.08%)**
  Dissolve 0.4 g of NH_4_Cl in 500 mL of bi-distilled water. Filter using a vacuum filtration system with 0.22 μm CA membrane and store at 4 °C.
4. **Kidney progenitor cell freezing medium**
  Immediately before freezing the cells, prepare a solution containing HyClone defined FBS supplemented with 10% DMSO.
5. **DMEM-F12 + 10% HyClone defined FBS**
  Dissolve 15.6 g of powder DMEM-F12 (one vial of DMEM-F12) in 1 L of MilliQ water and supplement with 1.2 g/L NaHCO_3_. Filter using 0.22 μm filters and store at 4 °C. Add FBS HyClone at a final concentration of 10% (w/v) only for the volume of medium necessary for the experiment.
6. **FACS BUFFER (PBS 1× 0.5% BSA - 0.02% NaN_3_ buffer)**
  Dissolve 2 g of NaN_3_ in 10 mL of PBS to obtain a 20% (w/v) NaN_3_ stock solution, which can be stored at room temperature for at least two years.
  Dissolve 1.25 g of BSA in 250 mL of D-PBS, no calcium, no magnesium, and then add 250 μL of 20% NaN_3_ stock solution. Store at 4 °C.
